# Supplementary material for: A disease associated mutant reveals how Ltv1 orchestrates RP assembly and rRNA folding of the small ribosomal subunit head
Source: bioRxiv. 2023 Jul 10:2023.07.10.548325. Preprint. [Version 1] doi: 10.1101/2023.07.10.548325 (PMC10369890; doi:10.1101/2023.07.10.548325)
Supplement: Supplement 1 [file NIHPP2023.07.10.548325v1-supplement-1.pdf]

bioRxiv preprint doi: https://doi.org/10.1101/2023.07.10.548325; this version posted July 10, 2023. The copyright holder for this preprint (which was not certified by peer review) is the author/funder, who has granted bioRxiv a license to display the preprint in perpetuity. It is made available under aCC-BY 4.0 International license.

Supplementary Materials for:

A disease associated mutant reveals how Ltv1 orchestrates RP assembly and quality control across the small subunit head

Ebba K. Blomqvist[1], Haina Huang[1,2,3] and Katrin Karbstein[1,2*]

bioRxiv preprint doi: https://doi.org/10.1101/2023.07.10.548325; this version posted July 10, 2023. The copyright holder for this preprint (which was not certified by peer review) is the author/funder, who has granted bioRxiv a license to display the preprint in perpetuity. It is made available under aCC-BY 4.0 International license.

Supplementary Figures and Legends:

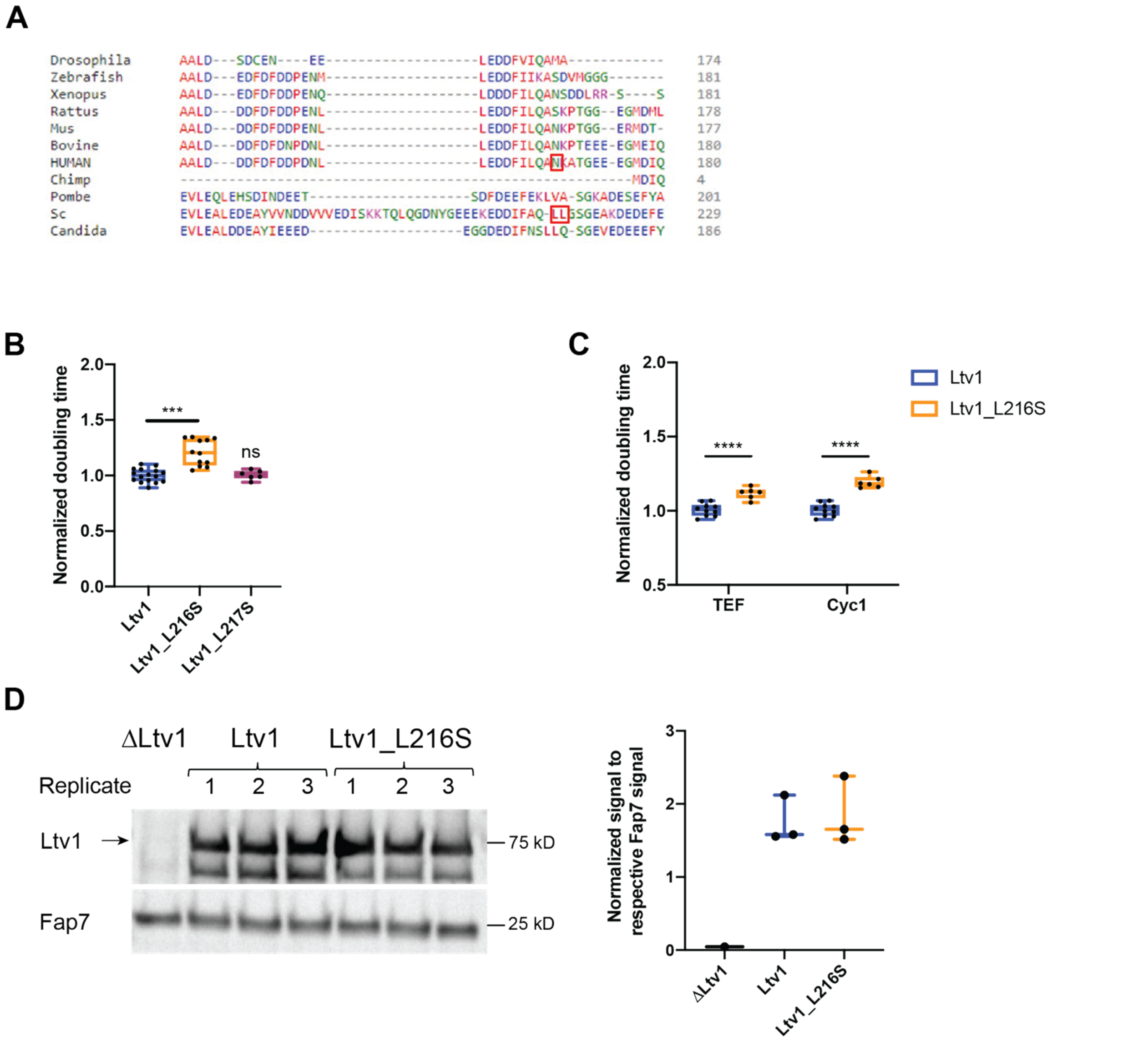

Figure S1: Yeast Ltv1_L216S corresponds to human Ltv1_N168S. (Relates to Figure 2) (A) Sequence alignment of a portion of Ltv1 highlighting N168 and L216 in human and yeast Ltv1, respectively. (B) Normalized doubling time of yeast lacking endogenous Ltv1 and expressing either wt Ltv1, Ltv1_L216S or Ltv1_L217S from TEF-promoter-driven plasmids. Significance was tested using an unpaired t-test. ***, P<0.001. Note that the data for WT Ltv1 and Ltv1_L216S are the same as in Figure 1B. (C) Normalized doubling time of yeast lacking endogenous Ltv1 and expressing either wt Ltv1, Ltv1_L216S or Ltv1_L217S from either TEF or Cyc1-promoter-driven plasmids. Significance was tested using an unpaired t-test. ****, P<0.0001. (D) Left: Western analysis of yeast lysates prepared from cells lacking endogenous Ltv1 and expressing either wt Ltv1 or Ltv1_L216S from TEF-promoter-driven plasmids. Three replicates for each are shown, as well as a control from ΔLtv1 cells. Fap7 is used as a loading control. Right: Quantification of the data on the left.

bioRxiv preprint doi: https://doi.org/10.1101/2023.07.10.548325; this version posted July 10, 2023. The copyright holder for this preprint (which was not certified by peer review) is the author/funder, who has granted bioRxiv a license to display the preprint in perpetuity. It is made available under aCC-BY 4.0 International license.

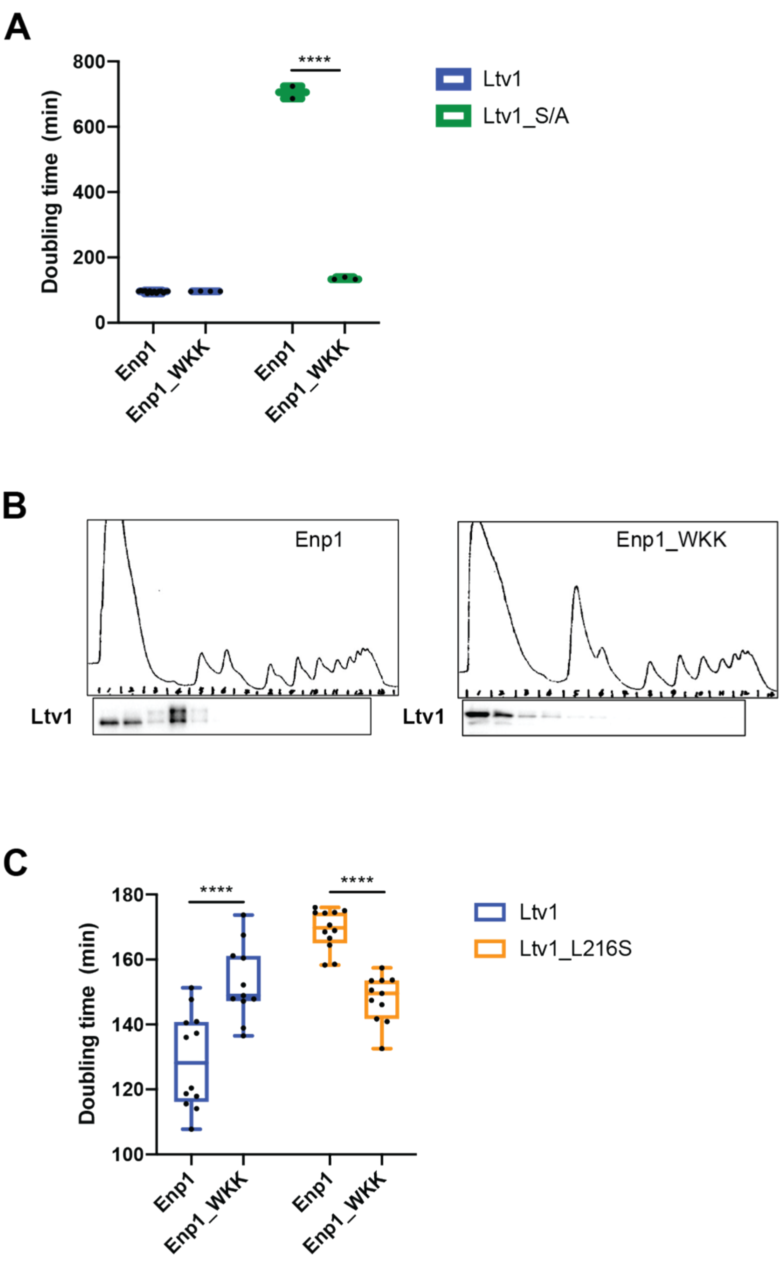

Figure S2: Enp1_WKK binds Ltv1 weakly, allowing for its phosphorylation independent release. (Relates to Figure 2) (A) Doubling time of yeast depleted for endogenous Enp1 and lacking endogenous Ltv1 and expressing either wt Enp1 or Enp1_WKK and wt Ltv1 or phosphorylation-deficient Ltv1_S/A from TEF-promoter-driven plasmids. Significance was tested using an unpaired t-test. ****, P<0.0001. (B) Absorbance profile (top) and Western blot (bottom) of yeast lysates from cells expressing either wt Enp1 or Enp1_WKK. (C) Doubling time of yeast depleted for endogenous Enp1 and lacking endogenous Ltv1 and expressing either wt Enp1 or Enp1_WKK and wt Ltv1 or Ltv1_L216S from TEF-promoter-driven plasmids. Significance was tested using an unpaired t-test. ****, P<0.0001.

bioRxiv preprint doi: https://doi.org/10.1101/2023.07.10.548325; this version posted July 10, 2023. The copyright holder for this preprint (which was not certified by peer review) is the author/funder, who has granted bioRxiv a license to display the preprint in perpetuity. It is made available under aCC-BY 4.0 International license.

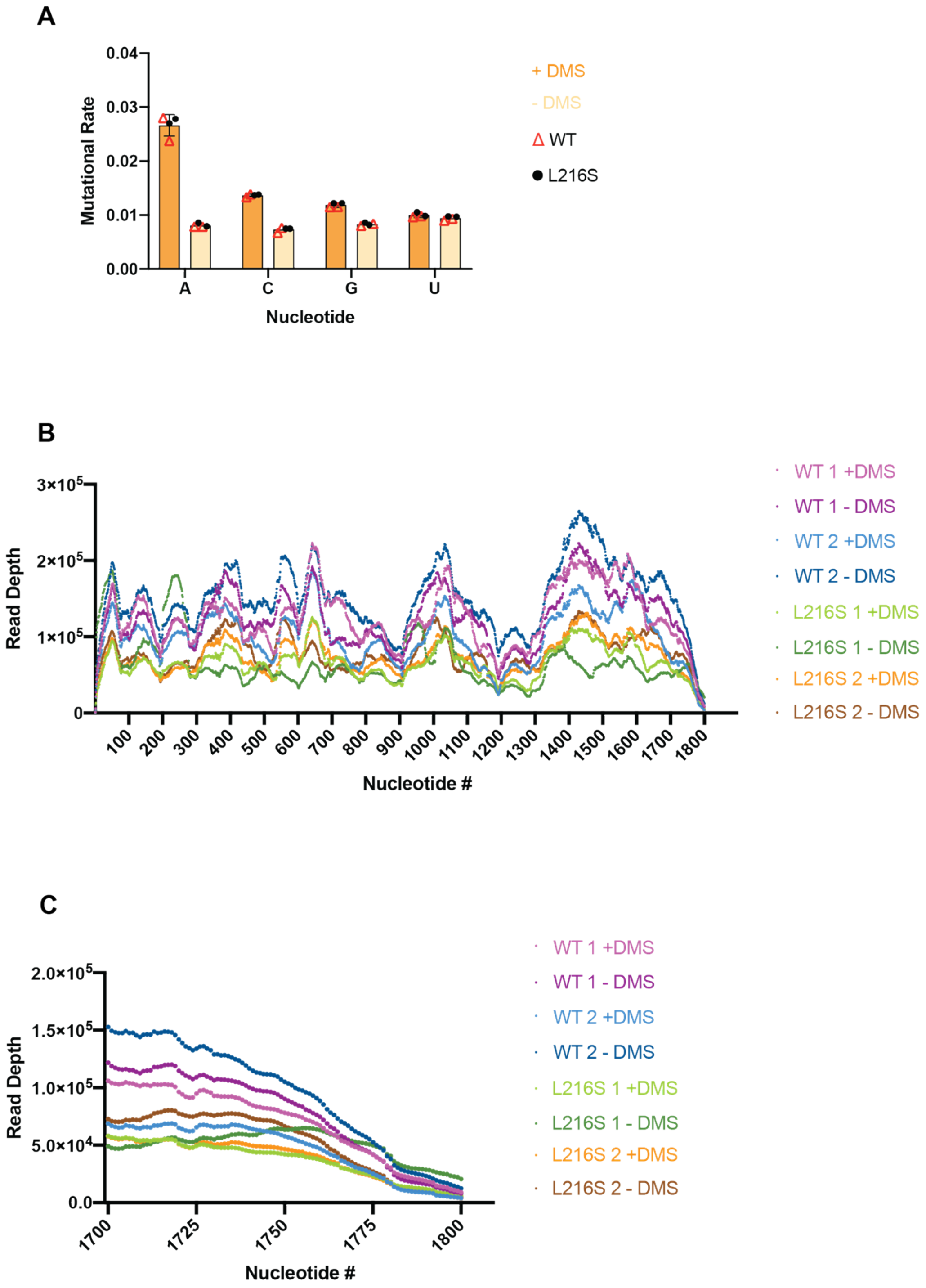

Figure S3: DMS-MaPseq quality control. (Relates to Figure 6). (A) Mutational rate changes for A and C residues upon DMS addition. Note that G also does get modified by DMS. (B) High read depth over the entire molecule. (C) Mature 18S rRNA is captured in all samples.

bioRxiv preprint doi: https://doi.org/10.1101/2023.07.10.548325; this version posted July 10, 2023. The copyright holder for this preprint (which was not certified by peer review) is the author/funder, who has granted bioRxiv a license to display the preprint in perpetuity. It is made available under aCC-BY 4.0 International license.

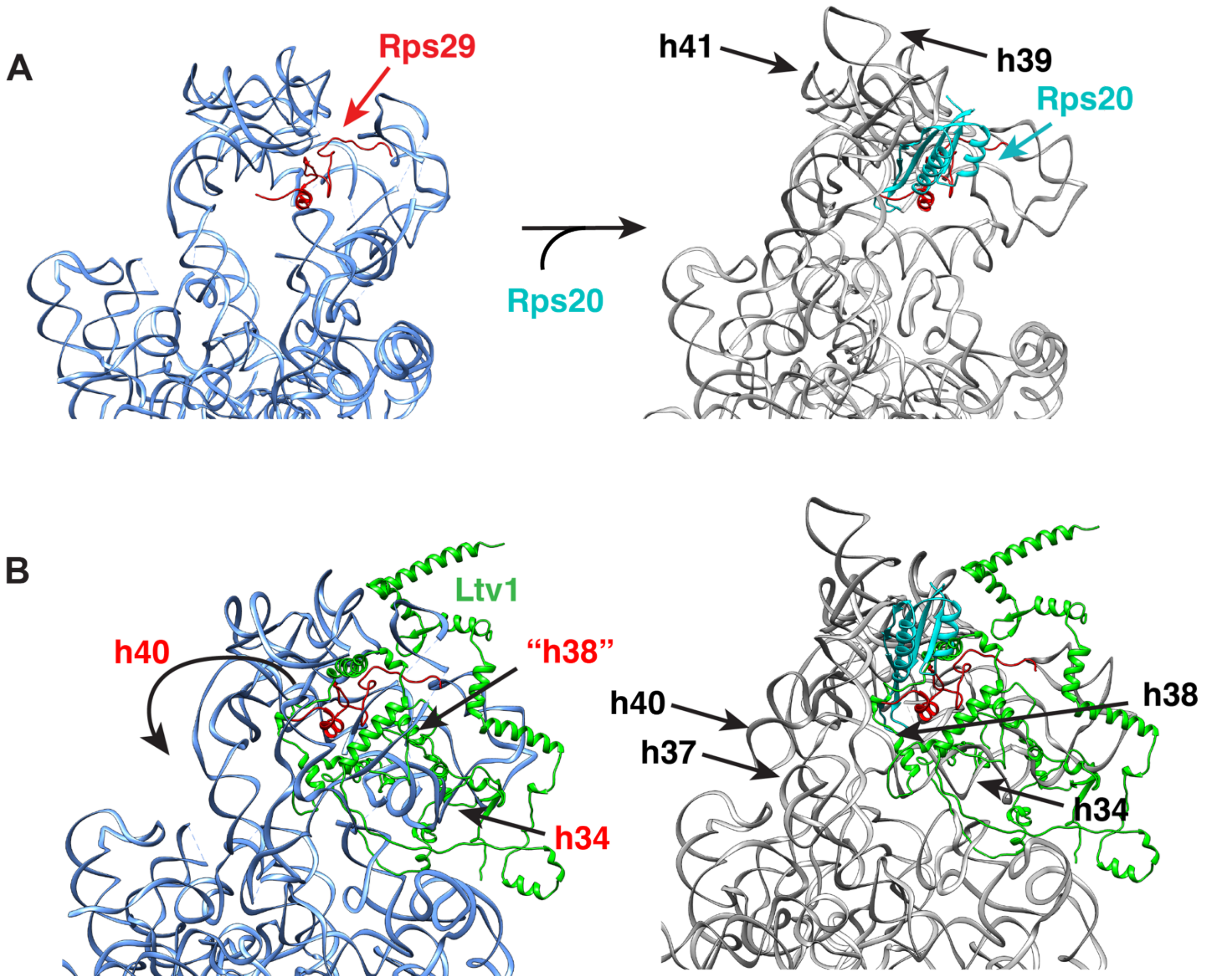

Figure S4: Binding of Rps20 is coupled to folding of h39 (Relates to Figure 6). (A) Structural detail of the 40S head (from PDB ID 7WTT) before and after Rps20 binding (from PDB ID 6FAI). For clarity only Rps20 and Rps29 are shown. (B) Structural details of the two 40S assembly intermediates in panel A, illustrating the sequential formation of helicase around j34-35-38 and the binding of Ltv1 adjacent to one strand of h38.

bioRxiv preprint doi: https://doi.org/10.1101/2023.07.10.548325; this version posted July 10, 2023. The copyright holder for this preprint (which was not certified by peer review) is the author/funder, who has granted bioRxiv a license to display the preprint in perpetuity. It is made available under aCC-BY 4.0 International license.

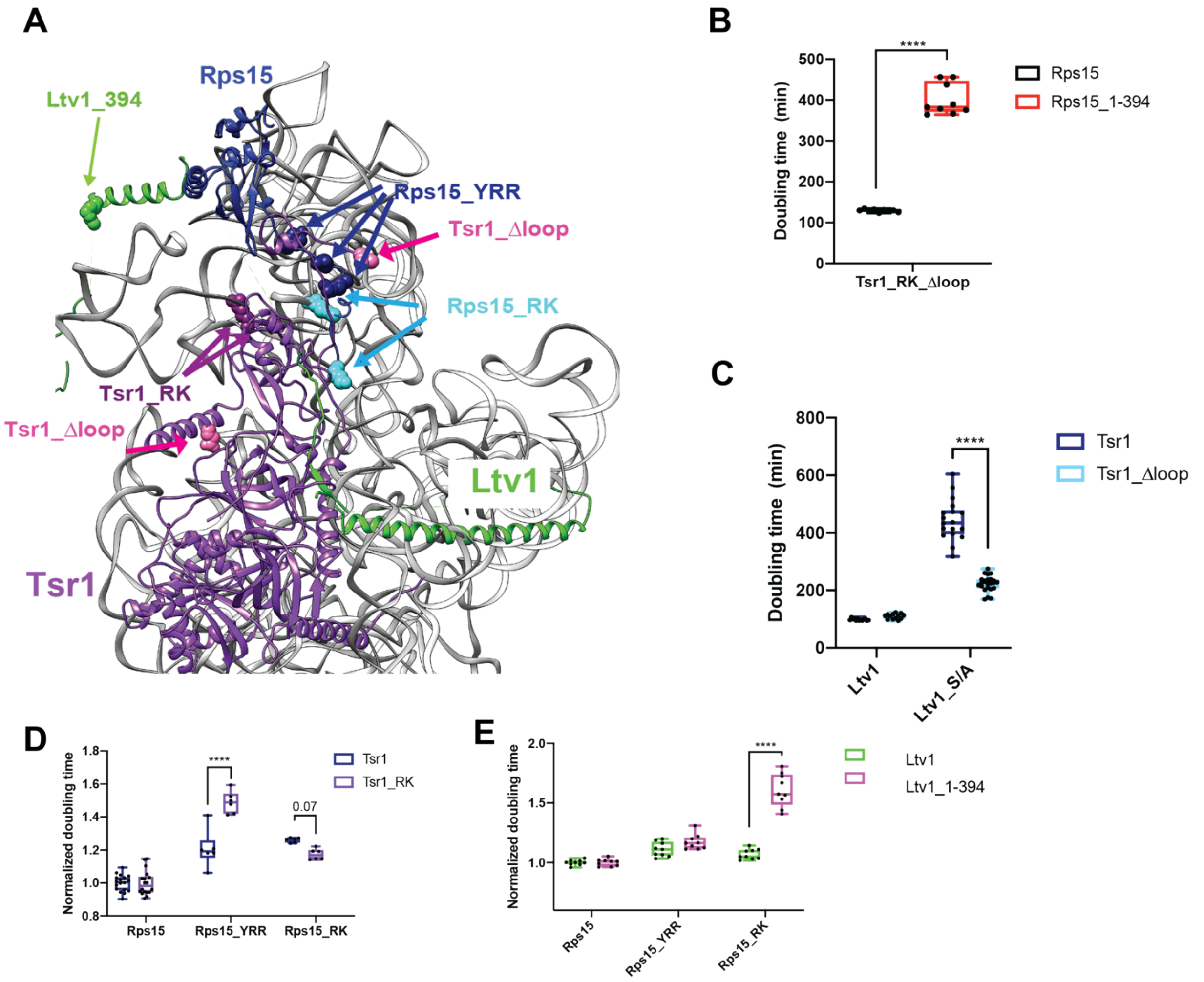

Figure S5: A structural and genetic network between Ltv1, Rps15 and Tsr1 (Relates to Figure 7). (A) Structural detail of a composite structure of yeast pre-40S (PDB:6FAI) and human Ltv1 (PDB ID 6G18) highlighting elements in Tsr1, Rps15 and Ltv1. The residues mutated in Rps15_YRR and Rps15_RK are shown in blue and cyan spheres, respectively. The residues mutated in Tsr1_RK are shown in magenta spheres, and the first and last amino acids of the loop removed in Tsr1_Δloop are shown in magenta. The last amino acid in the Ltv1 truncation Ltv1_1-394 is shown in green space fill. (B) Doubling times for yeast cells expressing either wt Ltv1 or Ltv1_3-194, and Tsr1_RK_Δloop. Data for WT Ltv1 and Tsr1_RK_Δloop are in [21]. Significance was tested using an unpaired t-test. ****, P<0.0001 (C) Doubling times for yeast cells expressing either wt Ltv1 or the phosphorylation-deficient Ltv1_S/A and either wt Tsr1 or Tsr1_Δloop. Significance was tested using an unpaired t-test. ****, P<0.0001. (D) Doubling times (normalized to wt Rps15) for yeast cells expressing either wt Rps15, Rps15_YRR or Rps15_RK and wt Tsr1 or Tsr1_RK. Significance was tested using an unpaired t-test. ****, P<0.0001. (E) Doubling times (normalized to wt Rps15) for yeast cells expressing either wt Rps15, Rps15_YRR or Rps15_RK and wt Ltv1 or Ltv1_1-394. Significance was tested using an unpaired t-test. ****, P<0.0001.

bioRxiv preprint doi: https://doi.org/10.1101/2023.07.10.548325; this version posted July 10, 2023. The copyright holder for this preprint (which was not certified by peer review) is the author/funder, who has granted bioRxiv a license to display the preprint in perpetuity. It is made available under aCC-BY 4.0 International license.

Table S1: Residues with altered DMS accessibility.

Residue No.	Average WT Ltv1	Average Ltv1_L216S	Δ(Ltv1_L216S vs. WT)	Change
100 (A)	0.9	1.6	0.7	2 bins, 1 bin
103 (A)	0.5	1.5	1.0	2 bins
172 (C)	0.5	1.8	1.2	1 bin
184 (C)	0.3	2.2	1.9	2 bins
191 (C)	5.1	2.7	-2.4	2 bins, 1 bin
221 (A)	0.5	2.7	2.2	2 bins
437 (A)	0.5	1.7	1.2	2 bins
990 (C)	2.4	4.1	1.7	1 bin, unchanged
1189 (A)	0.4	1.4	0.9	2 bins
1196 (A)	0.6	1.4	0.8	1 bin, unchanged
1197 (C)	0.8	2.0	1.2	2 bins
1505 (A)	3.4	4.2	0.8	1 bin, unchanged
1515 (A)	1.2	2.2	1.0	1 bin, unchanged
1591 (C)	0.6	2.4	1.8	2 bins, 1 bin
1753 (A)	1.6	3.3	1.7	2 bins, 1 bin

bioRxiv preprint doi: https://doi.org/10.1101/2023.07.10.548325; this version posted July 10, 2023. The copyright holder for this preprint (which was not certified by peer review) is the author/funder, who has granted bioRxiv a license to display the preprint in perpetuity. It is made available under aCC-BY 4.0 International license.

Table S2: Yeast strains used in this study.

Strains	Description	Genotype	Reference
YKK73	ΔLtv1	BY4741 (MATa His3-1 Leu2-0 Met15-0 Ura3-0), Ltv1::KAN	[25]
YKK422	ΔLtv1,Gal:Fap7	BY4741, Ltv1::KAN, Gal:Fap7(NAT)	[46]
YKK1230	ΔLtv1, Gal:S12	BY4741, Ltv1::KAN; Gal:Rps12 (NAT)	This work
YKK1389	ΔLtv1, snR35	BY4741, Ltv1::NAT; snR35 (KAN)	[22]
YKK1576	NOY504, ΔLtv1	W303a (*MATα, leu2-3*, *112*, *ura3-1*, *trp-1*, *his3-11*, *CAN1-100); rpa12*::*LEU2*, *Ltv1::HYG*	This work
YKK1115	ΔLtv1, Gal:S29	BY4741, Ltv1::HYG; Rps29B::KAN; Gal:3HA-Rps29 (NAT)	This work
YKK729	ΔLtv1,Gal:S20	BY4741, Ltv1::KAN, Gal:Rps20 (HYG)	[47]
YKK762	ΔLtv1,Gal:S3	BY4741, Ltv1::KAN, Gal:Rps3 (NAT)	[47]
YKK1117	ΔLtv1,Gal:S15	BY4741, Ltv1::KAN, Gal:Rps15 (NAT)	This work
YKK1625	ΔLtv1,Gal:S31	BY4741, Ltv1::NAT, Gal:Rps31 (HYG)	This work
YKK1190	ΔLtv1,Gal:Rio2	BY4741, Gal:Rio2(KAN), Ltv1::HYG	This work
YKK1142	ΔLtv1,Gal:Enp1	BY4741, Ltv1::KAN,Gal:Enp1(NAT)	[47]
YKK642	ΔLtv1,Gal:Tsr1	BY4741, Ltv1::KAN,Gal:Tsr1(NAT)	This work
YKK1184	Gal:Rio2,Gal:Tsr1	BY4741, Gal:Rio2(KAN), Gal:Tsr1(NAT)	[21]
YKK1444	Gal:Rps31,Gal:Tsr1	BY4741, Gal:31(HYG), Gal:Tsr1(KAN)	This work

bioRxiv preprint doi: https://doi.org/10.1101/2023.07.10.548325; this version posted July 10, 2023. The copyright holder for this preprint (which was not certified by peer review) is the author/funder, who has granted bioRxiv a license to display the preprint in perpetuity. It is made available under aCC-BY 4.0 International license.

Table S3: Plasmids used in this study.

Plasmid	Description	Vector	Reference	Detailed description
PKK3350	WT Rio2	TEF 413	[21]	
PKK3795	Rio2_K105E	TEF 413	[21]	K105E; see also [48]
PKK30356	Rio2_loop	TEF 413	[21]	R129A, H133A, R136A, R139A, D140A, K143A, K144A; see also [48]
PKK3295	WT Tsr1	TEF 416	[31]	
PKK3716	Tsr1_RK	TEF 416	[21]	R709E,K712E
PKK3895	Tsr1_Δloop	TEF 416	[21]	Substitution of amino acids 410 to 476 with PSSGSS; see also [49]
PKK30183	WT Tsr1	TEF 415	[21]	
PKK30184	Tsr1_RK	TEF 415	[21]	See PKK 3716
PKK30116	S15	TEF 416	[21]	
PKK30180	S15_YRR	TEF 416	[21]	Y123I,R127K,R130K
PKK30181	S15_RK	TEF 416	[21]	R137E,K142E
PKK3890	WT S20	TEF 415	[47]	
PKK3934	S20_DE	TEF 415	This work	D113A,E115A
PKK3891	S20_EYER	TEF 415	[47]	E80K,Y82A,E83K,R85E
PKK3848	TET S20	pCM189	This work	Tet-off Rps20
PKK3606	WT Ltv1	TEF 413	[46]	
PKK3607	Ltv1_S/D	TEF 413		Ltv1_S336D,S339D,S342D
PKK30749	Ltv1_L216S	TEF 413	This work	Ltv1_L216S
PKK30753	Ltv1_L217S	TEF 413	This work	Ltv1_L217S
PKK30643	Ltv1	CYC 415	This work	
PKK30762	Ltv1_L216S	CYC 415	This work	
PKK3693	Ltv1_ΔC394	TEF 413	[21]	Deletion of residues after 394.
PKK30750	Ltv1_L216S_S/D	TEF 413	This work	Ltv1_L216S and Ltv1_S336D,S339D,S342D
PKK30752	Ltv1_L216S_ΔC394	TEF 413	This work	Ltv1_L216S and deletion of residues after 394.
PKK30266	Ltv1_YDY	TEF 413	This work	Ltv1_Y82A,D83R,Y84A
PKK31167	S31	GPD 416	This work	
PKK31168	S31_Δubi	GPD 416	This work	Deletion of the N-terminal ubiquitin
PKK31169	S31_ ΔN	GPD 416	This work	Deletion of the N-terminal extension, has ubiquitin
PKK3521	WT S3	TEF 416	[47]	
PKK3867	S3_KK	TEF 416	This work	S3_K7A,K10A
PKK3705	S3_KR	TEF 416	This work	S3_K75E, R76E
PKK4015	TET Rps3	pCM189	[50]	Tet-off Rps3
PKK30407	WT S12	TEF 415	This work	
PKK31165	S12_R108	TEF 415	This work	
PKK3944	WT S29	TEF 416	This work	
PKK3955	TET S29	pCM189	This work	Tet-off Rps29

bioRxiv preprint doi: https://doi.org/10.1101/2023.07.10.548325; this version posted July 10, 2023. The copyright holder for this preprint (which was not certified by peer review) is the author/funder, who has granted bioRxiv a license to display the preprint in perpetuity. It is made available under aCC-BY 4.0 International license.

PKK3541	WT Enp1	TEF 416	[21]	
PKK30159	Enp1_WKK	TEF 416	This work	Enp1_W224V,K228E,K231E
PKK3234	WT 18S	pJV12	[51]	
PKK30582	18S_A1285A	pJV12	[21]	
PKK31082	18S_A1286A	pJV12	This work	
PKK30586	18S_A1288U	pJV12	This work	
PKK30974	18S_A1422U	pJV12	This work	
PKK30585	18S_G1288C	pJV12	[21]	
PKK30640	18S_C1327G	pJV12	[21]	
PKK30641	18S_G1288C+C 1327G	pJV12	[21]	
